# Covalent Organic Frameworks-Based Electrochemical Sensors for Food Safety Analysis

**DOI:** 10.3390/bios13020291

**Published:** 2023-02-17

**Authors:** Zhenyu Lu, Yingying Wang, Gongke Li

**Affiliations:** 1 College of Chemistry and Chemical Engineering, Henan Institute of Science and Technology, Xinxiang 453000, China; 2 School of Chemistry, Sun Yat-sen University, Guangzhou 510006, China

**Keywords:** covalent organic frameworks, electrochemical sensors, food safety, antibiotics, pesticides, heavy metal ions

## Abstract

Food safety is a key issue in promoting human health and sustaining life. Food analysis is essential to prevent food components or contaminants causing foodborne-related illnesses to consumers. Electrochemical sensors have become a desirable method for food safety analysis due to their simple, accurate and rapid response. The low sensitivity and poor selectivity of electrochemical sensors working in complex food sample matrices can be overcome by coupling them with covalent organic frameworks (COFs). COFs are a kind of novel porous organic polymer formed by light elements, such as C, H, N and B, via covalent bonds. This review focuses on the recent progress in COF-based electrochemical sensors for food safety analysis. Firstly, the synthesis methods of COFs are summarized. Then, a discussion of the strategies is given to improve the electrochemistry performance of COFs. There follows a summary of the recently developed COF-based electrochemical sensors for the determination of food contaminants, including bisphenols, antibiotics, pesticides, heavy metal ions, fungal toxin and bacterium. Finally, the challenges and the future directions in this field are discussed.

## 1. Introduction

Food safety is one of the issues of most global concern because safe food is essential for promoting human health and sustaining life [1,2]. The World Health Organization has listed food safety as one of its top 11 priorities [3]. However, with population growth, food industrialization and trade globalization, the types and quantities of food components or contaminants may increase at each stage of food production, such as crop cultivation, food production, processing, packaging, transport and storage. With the frequent occurrence of food pollution incidents around the world, food analysis, as a predominant step in food quality and safety control, has received unprecedented attention [4]. However, food analysis has faced significant challenges due to the complexity of the food sample matrix, the variety of potential interferents and the uncertainty of contaminants [5,6]. Therefore, food analysis techniques should be continuously improved to meet the needs of food testing.

In recent years, analytical techniques such as high-performance liquid chromatography [7], gas chromatography coupled with mass spectrometry [8], microfluidics [9], enzyme-linked immunosorbent assay [10], fluorescence spectroscopy [11] and nuclear magnetic resonance [12] have been performed for food quality and safety analysis. These techniques are often sensitive, accurate and selective. However, there are some drawbacks, including long analysis times, expensive or complex instrumentations and the need for skilled operators [13,14]. In addition, the food industry is more inclined to use efficient technologies to monitor food safety and quality levels. Hence, there is an urgent need to develop an accurate, simple, cost-effective, portable and fast response technique for food safety analysis. In order to meet these requirements, an electrochemical sensor has been proposed by researchers [15].

An electrochemical sensor is an electronic instrument that can conduct quantitative detection of analytes through a redox process. The electrochemical sensor is usually composed of three electrodes: working, reference and counter electrodes [16]. These three electrodes are placed in a cell containing an electrolytic solution and an analyte, and the analyte will undergo a redox reaction on the working electrode surface and generate an electrical signal proportional to the concentration of the analyte [17]. Therefore, components or contaminants in food can be immediately recognized by the electrochemical sensors. It should be mentioned that the traditional bare electrode is usually limited in terms of sensitivity and selectivity [18]. Thus, various materials have been synthesized and used as a working electrode material to improve the selectivity, sensitivity and speed of the electrochemical sensor [19,20]. Among those materials, porous materials have attracted great attention due to properties such as large surface area, many active sites, good chemical stability and fast electron transfer ability [21].

Covalent organic frameworks (COFs), as a new kind of organic porous polymer, are connected to light elements (C, H, N, B) via covalent bonds [22,23]. COFs have attracted more and more attention due to their low density, good chemical stability, permanent porosity and specific surface area [24]. Recently, COFs have been widely used in electrochemical sensing because they can improve the selectivity, sensitivity and speed of electrochemical sensors [25]. For example, COFs can prevent the agglomeration of electroactive molecules owing to their highly ordered pore structure and adjustable functional groups [26]; Moreover, they can improve the stability and reliability of the constructed electrochemical sensor [27]. However, there are still some problems that urgently need to be solved in the application of COFs to electrochemical sensing.

**Figure 1:**
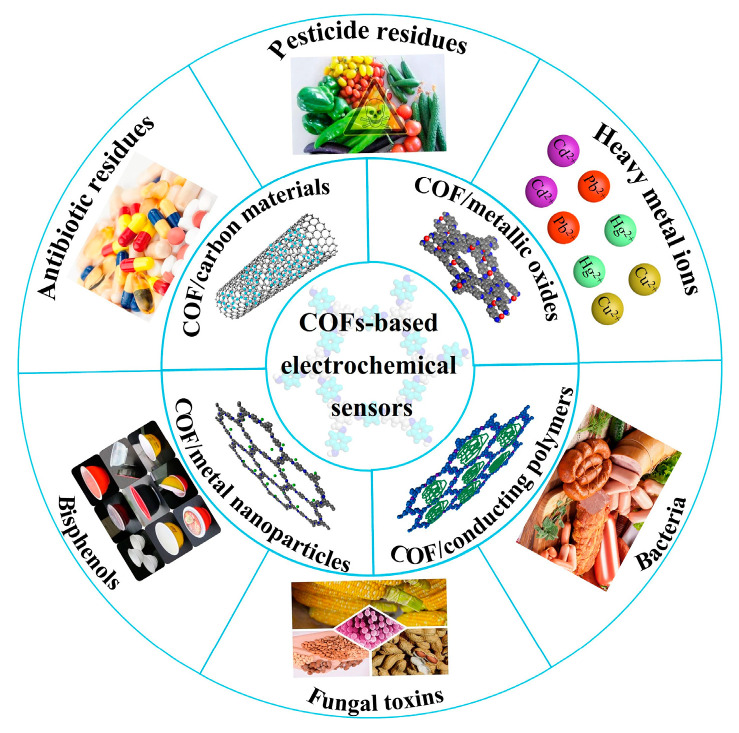
COF-based electrochemical sensors for food safety analysis.

This review aims to present the recent advances of COF-based electrochemical sensors which have been reported regarding their application to food safety analysis (Figure 1). The current synthesis methods of COFs and the strategies to improve their conductivity are discussed below in detail. Then, the sensors used for food contaminant determination, including the linear range, limit of detection (LOD) and real samples, are summarized and compared. Finally, the challenges and future prospects of these electrochemical sensors in food analysis are discussed.

## 2. Preparation of COFs and Improvement to Their Electrochemistry Performance

COFs have received extensive attention due to their wide application. Thus, it is critically important to develop new synthesis technologies to improve their application range. Recently, COFs have become an attractive material in electrochemical sensing because they can improve the selectivity, sensitivity and speed of electrochemical sensors [28]. Nevertheless, the poor intrinsic conductivity and weak electrocatalysis of most COFs obviously limit their application [29]. Therefore, the combination of COFs and materials with good conductivity is an ideal method to improve the electrochemical performance of COFs.

### 2.1. Preparation of COFs

Up to now, researchers have reported many synthesis approaches for COFs; five typical synthesis approaches—solvothermal synthesis, mechanochemical synthesis, solvent-free synthesis, microwave-assisted synthesis and sonochemical synthesis—are summarized (Table 1) and further discussed below.

#### 2.1.1. Solvothermal Synthesis

According to the reports, most of the prepared COFs are synthesized by the solvothermal synthesis method. In this method, monomers and solvents are added together in a Pyrex tube. After a freeze–pump–thaw cycle, the Pyrex tube is sealed and placed in an oven. The reaction often requires several days (2–9 d) under a controlled temperature (80–200 °C). After cooling to room temperature, COF is obtained through filtration, washing and drying. It should be pointed out that the solvent combinations and ratios and the reaction time have a great impact on the crystallinity and porosity of COFs in this method [56]. Since Omar M. Yaghi et al. [30] first reported the solvothermal synthesis of boronate ester-linked COF1 and COF5 in 2005, researchers synthesized more bonding types of COFs through this method, including imine- [31], olefin- [32], aminal- [33], thiazole- [34], sp^2^-carbon- [35] and polyimide -linked COFs [36]. Although the quality of COFs synthesized by the solvothermal method is often satisfactory, they also have limitations such as a long reaction time and organic solvents, which makes it difficult to expand them to industrial applications.

#### 2.1.2. Mechanochemical Synthesis

Mechanochemical synthesis has become an alternative synthesis method for preparing COFs due to its being simple, timesaving, solvent-free and operable at room temperature. Rahul Banerjee et al. [37] developed this method to produce a COF, named TpPa-1. Monomers 1, 3, 5-triformylphloroglucinol and *p*-phenylenediamine were placed in a mortar and ground for 5 min at room temperature, and then a COF with a light yellow color was obtained. Subsequently, several COFs such as TP-COP [38], TpMA [39] and TpMaCON [40] were also synthesized by using this method. Although these examples demonstrate the feasibility of COFs using the mechanochemical synthesis method, the generalization of this method is still a challenge because of the limitations in building monomers. In addition, COFs obtained by this method usually display low surface areas and inferior crystallinity compared with those prepared by the solvothermal synthesis method.

#### 2.1.3. Solvent-Free Synthesis

The solvent-free synthesis method has emerged as a viable method to prepare COFs owing to its environmental protection, simple operation, low cost-effectiveness and large-scale preparation [57]. Zhenjie Zhang et al. [41] first reported the solvent-free synthesis of olefin-linked COF (NKCOF-10). Under solvent-free conditions, NKCOF-10 was obtained through a benzoic-anhydride-catalyzed aldol reaction of 2, 5-dimethylpyrazine and 1, 3, 5-triformylbenzene. Subsequently, Zhenjie Zhang’s group used a similar method to synthesize various types of COFs, such as vinylene-linked COF [42], isomeric benzobisoxazole-vinylene-linked COF [43], olefin-linked COF [44,45] and C=N-linked COF [46]. Compared to the solvothermal synthesis method, this method usually requires the addition of solid-state catalytics (benzoic anhydride, benzoic acid, et al.) to improve the crystallinity and yield of COFs. The COFs obtained by this method have the advantages of high crystallinity and porosity, but it also requires high temperature and pressure, which limits its industrial application.

#### 2.1.4. Microwave-Assisted Synthesis

The microwave-assisted synthesis method has received more attention due to its advantages of shorter reaction times, higher yields, environmental protection and lower energy consumption [58]. Andrew I. Cooper et al. first prepared boronate ester-linked COF5 using microwave as the heat source in 20 min [47]. More interestingly, COF5 obtained using the microwave method had larger Brunauer–Emmett–Teller surface area (2019 m^2^/g) than that prepared by the conventional solvothermal method. In addition to boronate ester-linked COFs, covalent triazine-based framework [48], melamine-based COF [49], enamine-linked COF [50], cationic COF [51] and imine-linked COF [52] have also been synthesized by this microwave-assisted method. Now, microwave-assisted synthesis has become a potential method for the industrial synthesis of COFs because it can achieve faster and cleaner synthesis.

#### 2.1.5. Sonochemical Synthesis

The sonochemical method is capable of facilitating the homogeneity of COFs and accelerating the crystallization rate due to its use of ultrasonic wave, which can produce strikingly high pressure and local temperature, as a heat source [59]. However, the COFs prepared by the sonochemical synthesis method are still in a nascent stage. COF5 was first obtained in a sonicator unit under an adjustable power output [53]. More interestingly, COF5 processed significantly smaller crystals (50–250 nm) than that obtained by the solvothermal method. In addition, several other COFs have been synthesized using the sonochemical method, for instance, seven previously reported COFs (COF-1-COF-7) and two new COFs (SonoCOF-8, SonoCOF-9) [54,55]. The crystallinity and porosity of these COFs are comparable to or better than those of the same materials made by the solvothermal method. Sonochemical synthesis has become a potential method for the large-scale preparation of COFs owing to its super-fast synthesis rate and significantly reduced energy consumption.

The methods discussed above are used to prepare COFs. Nevertheless, the pure COFs usually have poor electrical conductivity, which limits their applications in the field of electrochemistry. Thus, new strategies that can improve the conductivity of COFs are urgently needed for their further electrochemical sensing applications.

### 2.2. Strategies to Improve Electrochemistry Performance of COFs

**Figure 2:**
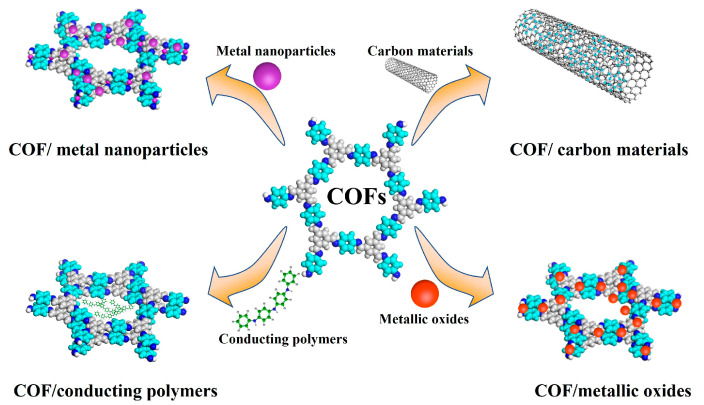
Strategies to improve COFs’ electrochemistry performance.

Combining COFs with some materials with specific conductivity, such as carbon materials, metal nanoparticles, metallic oxides and conducting polymers, can be considered an effective strategy to improve the electrochemistry performance of the COFs. Some of the strategies to enhance the COF electrical conductivity are described below (Figure 2 and Table 2):

#### 2.2.1. COF/Carbon Materials

Carbon-based materials have good conductivity, high specific surface area, excellent mechanical strength and cost effectiveness [74]. Thus, combining COFs with carbon materials has become a suitable solution to enhance the electron transfer of the COFs to meet the requirements of electrochemical sensing. Up to now, the conductive carbon materials used most widely include graphene [60,61], carbon nanotubes [62], fullerenes [63], macroporous carbon [64] and graphene aerogel [65]. All of these COF/carbon materials display more excellent electrochemical performance than COFs, carbon materials or glassy carbon electrode (GCE) alone. For example, Zhixiang Xu et al. [60] prepared a graphene oxide@COF composite modified by glassy carbon electrode (GO@COF/GCE). Electrochemical characteristics showed that the GO@COF/GCE had a larger peak current and electroactive surface area than the bare GCE due to the high surface area and good electrical conductivity of the GO@COF. A similar strategy was adopted by Dawei Pan et al. [61]. They synthesized a COF with a hydrogen sulfonic functional group. After the COF was combined with graphene, the composites showed excellent electrochemical performance. These studies confirmed that the use of COFs/carbon materials is an effective method in the construction of excellent electrochemical sensors. However, the binding mechanism of COFs and carbon materials remains unclear. Therefore, a large amount of work is still necessary to analyze these problems.

#### 2.2.2. COF/Metal Nanoparticles

The integration of metal nanoparticles (NPs) into a COF framework is another innovative method to improve COFs’ electrochemical performance. Not only can COFs provide multiple functional active sites and a large surface area for metal nanoparticles to integrate, but they can also prevent metal nanoparticle aggregation to obtain satisfactory dispersion. In addition, metal nanoparticles can improve the conductivity of COFs. Thus, the COF/metal nanoparticle composites have attracted more and more attention in the electrochemical field [75]. One example was reported by P. Arul et al. [66]. A COF was prepared with *p*-Phenylenediamine and terephthalaldehyde, and then Ag nanoparticles were embedded into the COF. Finally, the Ag NPs-COF/GCE was obtained after the composite was deposited on the GCE surface. Due to the synergistic effect of the COF and AgNPs, the AgNPs-COF/GCE showed 1.7 and 1.5 times higher electrical conductivity than the bare GCE, COF/GCE, and AgNPs/GCE alone. A similar approach, described by Wu Yang et al. [67], consisted of using Pt nanoparticles together with COFs as the electrode materials. In this example, the obtained electrode showed excellent electrocatalytic properties compared with those modified by individual materials. These studies confirm that the prepared COF/metal nanoparticle material is an effective strategy to obtain efficient electrochemical sensors, but the high cost of metal nanoparticles limits their large-scale application.

#### 2.2.3. COF/Metallic Oxides

**Figure 3:**
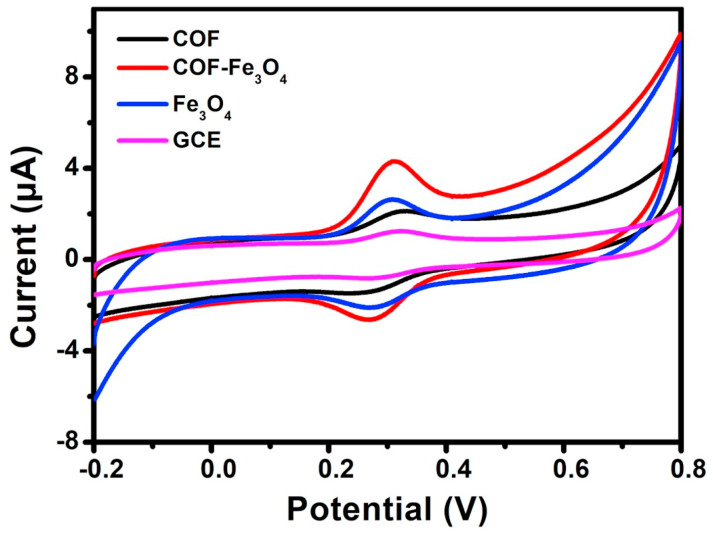
Cyclic voltammograms of Fe_3_O_4_ @TAPB-DMTP-COFs/GCE, bare GCE, TAPB-DMTP-COFs/GCE, and Fe_3_O_4_/GCE from Ref. [68]. Copyright 2020 Elsevier.

It is also an attractive approach to combine COFs with metal oxide nanomaterials to obtain hybrid materials for the development of electrochemical sensors. Until now, Fe_3_O_4_ [68], CuO [69], ZnO/ZnNi_2_O_4_ [70] and La_2_O_3_ [71] have been combined with COFs to finally construct electrochemical sensors. For example, Yang Wang et al. [68] prepared a core-shell structured magnetic COF (Fe_3_O_4_@TAPB-DMTP-COFs) by a step-by-step assembly method under facile conditions. The biggest peak current of luteolin on the TAPB-DMTP-COFs/GCE was observed on the Fe_3_O_4_@TAPB-DMTP-COFs/GCE (Figure 3), due to the synergistic effects of the high surface area of the TAPB-DMTP-COFs and the excellent catalytic activity of Fe_3_O_4_. Another example of the synthesis of COF/metal oxide composites was reported by Yang Wang et al. [69]. They prepared a CuO@TAPB-DMTP-COF composite with a core-shell structure by incorporating CuO nanorods into the TAPB-DMTP-COF framework. The electrochemical parameters showed that the electrical analytical performance of the TAPB-DMTP-COF/GCE was improved significantly owing to the synergistic effects of TAPB-DMTP-COF and CuO. These examples demonstrate that COF/metallic oxides are good candidates for preparing electrochemical sensors in various applications.

#### 2.2.4. COF/Conducting Polymers

**Figure 4:**
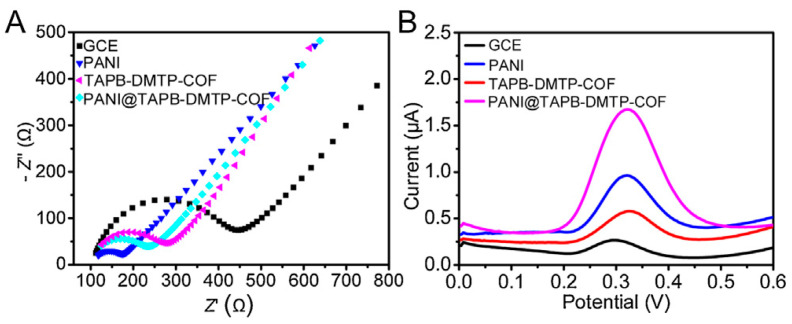
(**A**) EIS Nyquist plots and (**B**) Differential pulse voltammetry voltammograms of bare GCE, PANI/GCE, TAPB-DMTP-COF/GCE, and TAPB-DMTP-COF@PANI/GCE from Ref. [73]. Copyright 2021 Elsevier.

Another effective approach to improve the conductivity of COFs is to integrate the COFs and a conducting polymer. In the example described by William R. Dichtel et al. [72], in order to enhance the conductivity of a COF film, they encapsulated 3,4-ethylenedioxythiophene into COF pores by a facile electropolymerization method. The composite possessed significant improvement in involumetric energy and power densities relative to COF film alone. Moreover, the composite also had excellent cyclic stability. Another example for the preparation of a COF/conducting polymer composite was reported by Yi Wang et al. [73]. In that work, a core-shell structured COF/conducting polymer composite (TAPB-DMTP-COF@PANI) was synthesized using a simple polymerization approach. The electrochemical responses experiments showed that the TAPB-DMTP-COF@PANI/GCE displayed a stronger electrochemical signal compared to bare GCE, PANI/GCE, TAPB-DMTP-COF/GCE alone (Figure 4). Although only a few studies have been reported, it is still proved that COF/conducting polymer composites are promising functional materials to fabricate electrochemical sensors. Thus, more studies are necessary to expand the variety of these composites and further validate these findings.

Although the above strategies to improve the electrochemical properties of COFs have excellent performance, the types of conductive materials are relatively few. In the future, a variety of conductive composite materials need to be developed to meet the broad application of COF in the electrochemical field.

## 3. Applications in Food Safety Analysis

Based on their fascinating structures and properties, COFs have been successfully applied in the field of electrochemical sensing [76]. Nowadays, numerous food contaminants have been detected by COF-based electrochemical sensors, which have excellent selectivity, high sensitivity and quick response speed. An overview of these sensors for the determination of food contaminants, such as bisphenols, antibiotics, pesticides, heavy metal ions, fungal toxin and bacterium, is shown below in Table 3.

### 3.1. Bisphenols

Bisphenol compounds widely exist in plastic food packaging materials, and they have various adverse health effects on organisms, which will lead to serious diseases [99]. Therefore, it is necessary to develop a simple and sensitive method for bisphenol compound determination.

**Figure 5:**
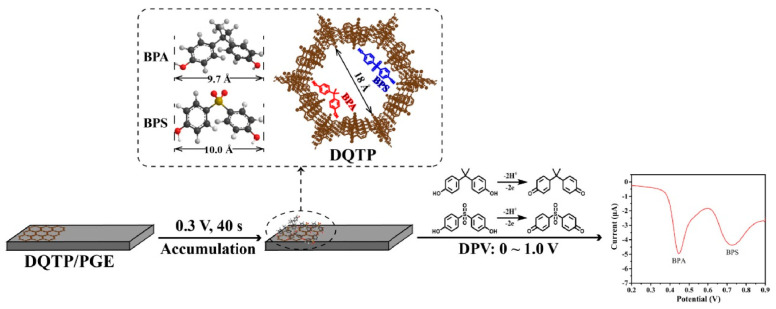
Schematic illustration of a DQTP-based electrochemical sensor for the simultaneous determination of BPA and BPS from Ref. [78]. Copyright 2022 Elsevier.

JinYu Qiao et al. [77] proposed a ratiometric electrochemical sensor for the simultaneous determination of BPA and BPS. The ratiometric sensor was prepared based on COF-LZU1 and silver nanoparticles modified with carbon cloth electrode (COF/AgNPs/CC). This electrode displayed excellent electrocatalytic activity on BPA and BPS due to its high electrocatalytic surface area and good conductivity. The LODs (S/N = 3) of the determination for BPA and BPS were both 0.15 μmol/L. Moreover, other electrochemical sensors based on COF (DQTP) modified with pencil graphite electrode (DQTP/PGE) (Figure 5) [78] and COF (CTpPa-2)-modified GCE [79] have also been established, and they have been successfully applied to BPA and BPS determination. Tert-butylhydroquinone (TBHQ) as one of a strong phenolic antioxidant is widely used in food products due to its excellent chemical stability and anti-lipid peroxidation property [80]. However, health studies indicated that excess intake of TBHQ would induce serious health issues towards humans. Recently, Yi Wang et al. [80] developed an electrochemical sensor based on core-shell Co_3_O_4_@TAPB-DMTP-COF composite for the sensitive and selective determination of TBHQ. The Co_3_O_4_@TAPB-DMTP-COF was synthesized using a monomer-mediated in situ growth strategy. In the electrochemical sensor, the COFs acted as a protective layer to accelerate current mobilization and the Co_3_O_4_ nanoparticles as the electrocatalytic active center. The LOD for the TBHQ determination was 0.002 μmol/L (S/N = 3).

The above studies prove that a COF-based electrochemical sensor can achieve the determination of bisphenol compounds. However, the current reports mainly involve bisphenol A and bisphenol S determination. Hence, it is necessary to design a novel electrochemical sensor based on COFs to determine more kinds of bisphenol compounds.

### 3.2. Antibiotics

Antibiotics, as a class of pharmaceuticals that can inhibit or kill pathogens, are widely used to prevent and treat infectious diseases caused by bacteria, fungi, molds and other microorganisms [100]. However, the excessive consumption of antibiotics can result in many health problems including blood dyscrasias, liver toxicity and allergic reactions [101].

**Figure 6:**
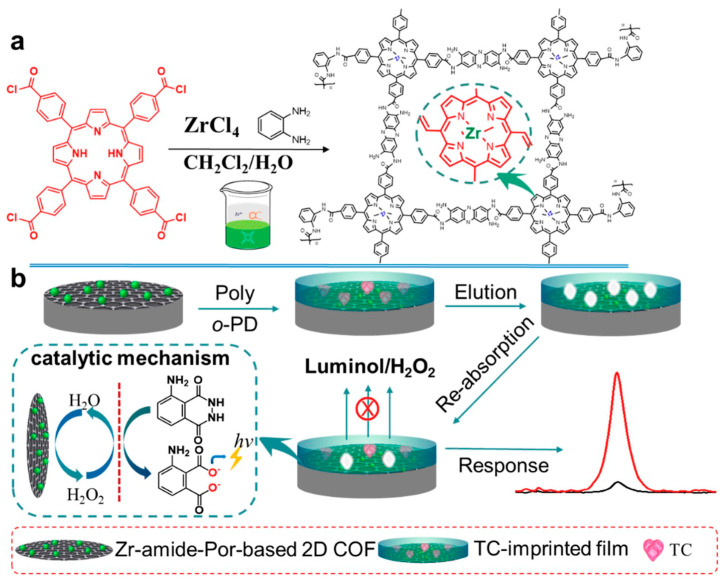
Schematic illustration of the synthesis of a Zr-amide-Por-based 2D COF and its application for the determination of TC from Ref. [82]. Copyright 2019 Elsevier.

Tetracycline (TC) is a typical antibiotic that can cause resistance and other side effects such as allergic reactions, nephrotoxicity and liver impairment. At present, some electrochemical sensors based on COFs have been used for tetracycline antibiotic detection. For example, Yukun Yang et al. [81] proposed a portable and on-site electrochemical sensor based on surface molecularly imprinted polymer (MIP)-modified magnetic COF (Fe_3_O_4_@COFs@MIPs) for the sensitive and rapid determination of TC. The Fe_3_O_4_@COFs@MIPs was synthesized according to a layer-by-layer modification method, which showed outstanding adsorption properties and good magnetism. The liner range of the electrochemical sensor for TC determination was 1 × 10^−10^–1 × 10^−4^ g/mL and the LOD was 2.4 × 10^−11^ g/mL (S/N = 3). The prepared sensor has also been successfully applied to milk and chicken samples. Xionghui Ma et al. [82] constructed an MIP electrochemiluminescence sensor based on a novel Zr- amide porphyrin-based 2D COF for TC determination in milk samples (Figure 6). The COF was obtained by a facile liquid–liquid interface method and showed remarkable electrocatalytic performance due to its inherently-ordered structure and abundant Zr catalytic active center. The LOD of the sensor for TC determination was 2.3 × 10^−6^ μmol/L. In another example, Hongming He et al. [83] synthesized a ferriporphyrin-based COF (Fe-PPOF) by a sonogashira coupling reaction. Then, an electrochemical aptasensor was constructed using aptamer-immobilized Fe-PPOF for oxytetracycline determination with an LOD of 2.05 fg/mL. Moreover, the electrochemical aptasensor has also been applied to the analysis of oxytetracycline in milk samples. Apart from TC, some other electrochemical sensors based on COFs have been reported for the determination of sulfamerazine [84], sulfadiazine [61], furazolidone [85], penicillin [86] and chloramphenicol [87].

### 3.3. Pesticides

**Figure 7:**
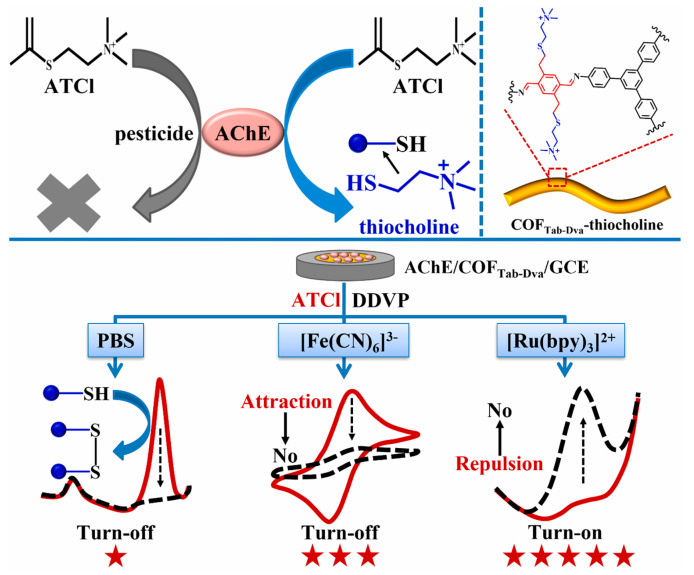
Schematic illustration of enzyme inhibition and DDVP determination from Ref. [90]. Copy right 2022 Elsevier.

Pesticide residues in food samples have become a great threat to human life. Thus, it is urgent to detect pesticide residues in a sensitive, selective, simple and rapid manner. Carbaryl is a carbamate pesticide that can cause damage to the nervous system and brain [102]. Lili Chen et al. [88] proposed a N, O-rich COF paper constructed-based electrochemical biosensor with repeatability, selectivity and stability for carbaryl detection. The COF was prepared through an aminealdehyde condensation reaction, which had abundant C=O, NH and OH groups. Then, the COF was fixed on a paper electrode to load acetylcholinesterase, which could greatly enhance the bioactivity of acetylcholinesterase. The COF was able to capture thiocholine by hydrogen bonding. The linear range of the biosensor was 0.39–35 μmol/L with an LOD of 0.13 μmol/L. The electrochemical biosensor has also been used for the determination of carbaryl in lettuce juice samples. In a similar example, Shuo Wang et al. [89] designed an MIP electrochemiluminescence sensor, based on COF and carbon nitride nanosheets, which can detect carbaryl sensitively and accurately. Organophosphorus pesticides are organic compound pesticides containing the phosphorus element; they are widely used in agricultural production, resulting in various degrees of residues in crops [103]. Guangran Ma et al. [90] reported a ratiometric electrochemical biosensor based on a COF_Tab-Dva_ nanofiber with excellent electroactivity for O, O-dimethyl-O-2, 2-dichlorovinylphosphate (DDVP) determination (Figure 7). COF_Tab-Dva_ nanofibers can effectively immobilize acetylcholine and enrich thiocholine due to their large surface area and abundant vinyl. The LOD of the electrochemical biosensors was 0.11 μmol/L. More interestingly, the ratiometric electrochemical biosensor was successfully used to detect the DDVP residual in lettuce juice samples. Xianbo Lu et al. [91] also synthesized a COF with abundant carbonyl groups and used the same to construct an electrochemical sensor for paraoxon determination in cucumber samples. In addition, Xue Wang [92] and Yonggui Song [93] have constructed COF-based electrochemical sensors for the detection of malathion and trichlorfon, respectively.

Although there are few reports on the application of COF-based electrochemical sensors in pesticide residue determination, they still prove that this sensor is a promising tool for pesticide determination. Therefore, more research is urgently needed to expand the variety of COF composites and pesticides to validate these findings.

### 3.4. Heavy Metal Ions

Heavy metals, such as Pb^2+^, Hg^2+^, Cu^2+^, and Cd^2+^, can accumulate in the food chain at different stages. These heavy metals have serious effects on human health even at ultra-trace levels [104]. Therefore, it is very important to detect heavy metals in food samples.

Tayyebeh Madrakian et al. [94] developed a novel simple, sensitive and rapid electrochemical sensor based on melamine-COF-modified glassy carbon electrode for the simultaneous measurement of Pb^2+^ and Hg^2+^. The linear range of the sensor was 0.01–0.3 and 0.05–0.3 μmol/L for Pb^2+^ and Hg^2+^, respectively, with LODs of 0.72 × 10^−3^ and 1.2 × 10^−2^ μmol/L. Moreover, the electrochemical sensor was also used to detect Pb^2+^ and Hg^2+^ in edible samples. Madrakian et al. [95] also proposed a glass carbon electrode modified by an electroplated bismuth film and a nanocomposite of melamine-COF and Fe_3_O_4_ nanoparticles. The glass carbon electrode successfully realized the selective detection of Pb^2+^ with a LOD of 0.95 nnmol/L. In order to achieve the simultaneous detection of multiple heavy metal ions, Yanyan Zhang et al. [96] reported a COF with calcium lignosulfonate-modified multiwalled CNTs and nafion (COF/MWCNTs/CLS) was used as an electrocatalytic material to modify GCE. The modified electrode has been used for the simultaneous detection of Cu^2+^, Pb^2+^ and Cd^2+^ due to the high enrichment capacity of the COF and the good conductivity of the MWCNTs. The LODs of the electrochemical sensor for Cu^2+^, Pb^2+^ and Cd^2+^ determination were 0.2, 0.7 and 0.4 μg/L, respectively.

### 3.5. Fungal Toxin and Bacterium

**Figure 8:**
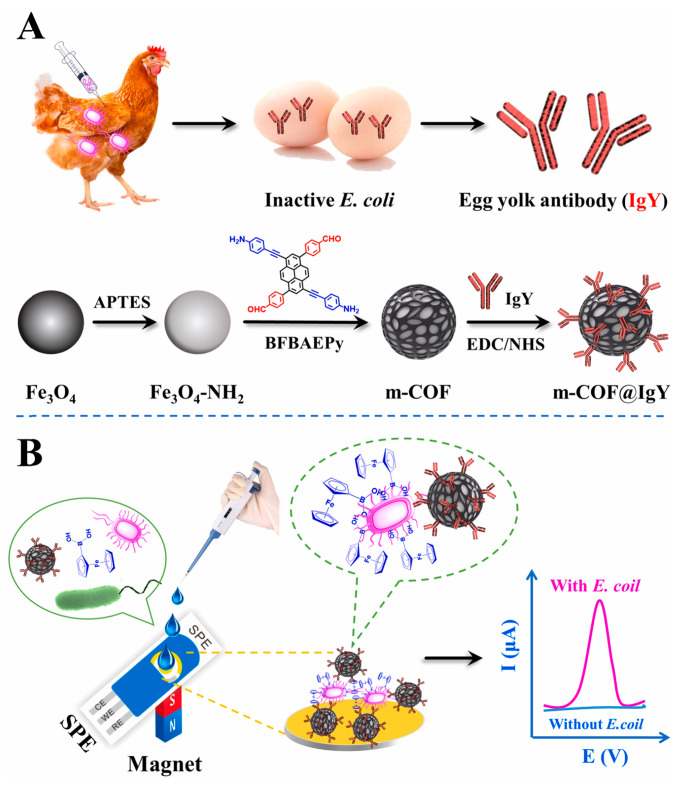
(**A**) Schematic illustration of the preparation process of m-COF@IgY and (**B**) application for determination of *E. coli* from Ref. [98]. Copyright 2022 Elsevier.

Aflatoxin is a metabolite of aspergillus flavus, aspergillus parasiticus and aspergillus norvegicus, which mainly exists in moldy peanuts, cereals and other foods [105]. Due to the high toxicity and carcinogenicity of aflatoxin, it is necessary to determine it in food. Recently, Yuehong Pang et al. [97] prepared a COF (TpBD) -modified GCE by a reliable in situ growth method. Then, the modified GCE was used as a working electrode for the electrochemical analysis of aflatoxin M1 in milk samples with magnetic separation technology. The proposed electrochemical sensor had a lower LOD of 0.15 ng/mL (S/N = 3) due to the large surface area of the COF. Bacterial contamination has attracted increasing attention because it can cause foodborne diseases. Thus, it is important for human health to use simple and rapid methods to monitor bacterial contamination. Ning Gan et al. [98] developed an electrochemical biosensor based on egg yolk antibody-labeled magnetic COF for the determination of Escherichia coli in milk, beef and shrimp samples (Figure 8). Magnetic COF, due to its large surface area and abundant holes, was used to label egg yolk antibodies so as to achieve the efficient enrichment and detection of *E. coli*. The linear range of the electrochemical biosensor was 10–10^8^ CFU/mL with the LOD of 3 CFU/mL.

Although only a few COF-based electrochemical sensors have been designed and applied to fungal toxin and bacterium analysis, it has still been proven that the COFs are a propagable material to construct an electrochemical sensor for fungal toxin and bacterium determination.

## 4. Conclusions

Food safety is one of the critically important issues for human health with the continuous growth of food consumption and more reports of food contamination incidents. To ensure food safety, electrochemical sensors have been employed for food quality and safety testing due to their accurate, simple, cost-effective and fast response. Herein, we have reviewed the reported COF-based electrochemical sensors in the context of food safety analysis. Specifically, the design and synthesis methods of COFs, namely solvothermal synthesis, mechanochemical synthesis, solvent-free synthesis, microwave-assisted synthesis and sonochemical synthesis, have been summarized. Moreover, these reports have been discussed in terms of the strategies to improve the performance of COF based electrochemical sensors as well as their use in the determination of food contaminants, including bisphenols, antibiotics, pesticides, heavy metal ions, fungal toxins and bacterium. Thus, COFs provide an emerging platform for researchers to develop electrochemical sensors for food safety applications. Although COF-based electrochemical sensors have demonstrated excellent performance and promising prospects, there are still some challenges, such as the need to strengthen the stability and repeatability of the sensors and to realize the miniaturization and field operation of the sensors. Although challenges still exist, the exciting promise of these sensors in the electrical analysis of food will encourage researchers to conduct further research, combining new COFs with advanced electrochemical techniques to give electrochemical sensors perfect analytical performance.

In general, the application of COF-based electrochemical sensors in the field of food analysis is still at an early stage, and there is still much room for research in this innovative field. Taking a future outlook, the rational design and controlled synthesis of multifunctional electrode materials based on COFs, while enhancing their selectivity to food contaminants, are very desirable for expanding their applications in the field of food analysis. In addition, the poor biocompatibility of COFs limits their potential application in contaminant detection in biological foods. In the future, the feasibility study of biocompatibility of electrochemical sensors based on COFs should be strengthened. The synthesis of new compatible/biocompatible COFs is very important for the analysis of contaminants in biological food. Furthermore, when detecting targets in complex systems, one of the main challenges faced by electrochemical sensors is the fouling of the electrode surface, which leads to the limited life of the sensors. This problem should be solved by designing and synthesizing COF electrode materials with antifouling performance.

## Figures and Tables

**Table 1:** The synthetic approaches of COFs.

Synthesis Methods	Energy	Time (min)	Temperature (°C)	Solvents	Advantages	Disadvantages	Refs.
Solvothermal synthesis	Oven heater	2–9 d	80–200	1,4-dioxane; acetic acid; TFA; Toluene; DMSO; *o*-DCB; EtOH; *m*-cresol; NMP; isoquinoline	The most commonly used synthesis method; High crystallinity	Long reaction time; require organic solvents	[30,31,32,33,34,35,36]
Mechanochemical synthesis	Mechanical force	5–300	RT	-	Simple, time-saving, solvent-free and operable at room temperature	Low surface areas and inferior crystallinity	[37,38,39,40]
Solvent-free synthesis	Oven heater	3–5 d	120–200	-	Environmental protection; High crystallinity	Requires solid state catalytics, high temperature and pressure	[41,42,43,44,45,46]
Microwave-assisted synthesis	Microwave radiation	30–360	80–110	TfOH; DMSO; Mesitylene; 1,4-dioxane; acetic acid	Less reaction time, higher yields, environmental protection and lower energy consumption	Low crystallinity	[47,48,49,50,51,52]
Sonochemical synthesis	Ultrasonic radiation	60–120	RT	Mesitylene; 1,4-dioxane; acetic acid	Fast synthesis rate and significantly reduced energy consumption; High crystallinity	Require high temperature	[53,54,55]

TfOH: trifluoromethanesulfonic acid; TFA: trifluoroacetic acid; DMSO: dimethylsulphoxide; *o*-DCB: 1,2-dichlorobenzene; EtOH: ethanol; NMP: N-Methylpyrrolidone; RT: room temperature; -: no needed.

**Table 2:** Methods to improve the electrochemistry performance of COFs and their advantages and disadvantages.

Methods	Advantages	Disadvantages	Refs.
COF/Carbon Materials	Large surface areas, abundant active sites and excellent conductivity	The binding mechanism remains unclear	[60,61,62,63,64,65]
COF/Metal Nanoparticles	Many kinds of metal nanoparticles; Faster electron transfer rate and excellent electrical conductivity	High cost of metal nanoparticles	[66,67]
COF/Metallic Oxides	Excellent conductivity and functionality; Large surface areas	Tedious preparation process	[68,69,70,71]
COF/Conducting Polymers	Simple preparation; Remarkable electrocatalytic performance	Few types of conductive materials	[72,73]

**Table 3:** COF-based sensors for food safety analysis.

Working Electrode	Samples	Techniques	Analytes	Linear Range (μmol/L)	LOD ^m^ (μmol/L)	Advantages	Disadvantages	Ref.
Bisphenols								
COF/AgNPs/CC ^a^	waters, tea, juice, beer	DPV ^e^	Bisphenol A	0.5–100	0.15	Better reproducibility, wider linear range and low LOD	The types of bisphenol compounds detected are limited	[77]
			Bisphenol S	0.5–100	0.15			
DQTP/PGE ^b^	Acidic food	DPV	Bisphenol A	0.5–30	0.15			[78]
			Bisphenol S	0.5–30	0.15			
CtpPa-2/GCE	Bottles	DPV	Bisphenol A	0.1–50	0.02			[79]
			Bisphenol S	0.5–50	0.09			
Co_3_O_4_@TAPB-DMTP-COF/GCE	Edible oil	DPV	Tert-butyl hydroquinone	0.05–1.0; 1.0–400	0.002			[80]
Antibiotics								
Fe_3_O_4_@COFs@MIPs/SPE ^c^	Milk, Chicken	DPV	Tetracycline	1 × 10^−10^–1 × 10^−4^ g/mL	2.4 × 10^−1^ g/mL	Excellent stability, superior anti-interference ability and can detect different types of antibiotics	It is difficult to realize simultaneous detection of multiple antibiotics	[81]
Zr-amide-Por-based 2D COF/GCE	Milk	ECL ^f^	Tetracycline	5 × 10^−6^–6 × 10^−5^	2.3 × 10^−6^			[82]
Fe-PPOF/AE ^d^	Milk	EIS ^g^	Oxytetracycline	2.2 × 10^−8^–1.09 × 10^−3^	4.45 × 10^−9^			[83]
MoS_2_/NH_2_-MWCNT@COF/GCE	Pork, chicken	DPV	Sulfamerazine	3.0 × 10^−4^–2.0 × 10^−1^	1.1 × 10^−4^			[84]
MIP/GO@COF/GCE	Beef and fodder	DPV	Sulfadiazine	0.5–200	0.16			[61]
COF@NH_2_-CNT/GCE	Chicken, lamb	DPV	Furazolidone	0.2–100	77.5 × 10^−3^			[85]
atp/POP/AE	Milk	EIS	Penicillin	0.001–10 mg/L	3.2 × 10^−4^ mg/L			[86]
Au@COF/GO-NH_2_/AE	Milk	EIS	Chloramphenicol	0.155–3.09 × 10^−3^	4.99 × 10^−8^			[87]
Pesticides								
AChE/COF_DHNDA-BTH_/GCE	Lettuce juice	CV ^h^	Carbaryl	0.48–35	0.16	Fast response, high sensitivity, good selectivity and practicability	Multiple pesticides cannot be analyzed at the same time	[88]
MIPs/DAFB-DCTP@CNNs/GCE	Milk, fruit wine	ECL	Carbaryl	1 × 10^−7^–50	4.67 × 10^−8^			[89]
AChE/COF_Tab-Dva_/GCE	lettuce	DPV	DDVP ^l^	0.33–30	0.11			[90]
GC/COF1/AChE/GCE	cucumber	CV	Paraoxon	10–1000 μg/L	1.4 μg/L			[91]
COF@MWCNTs/GCE	Spinach	DPV	Malathion	1 × 10^−3^–10	0.5 × 10^−3^			[92]
COF-LZU1/3D-KSCs	Schisandra chinensis	DPV	Trichlorfon	0.2–19 μg/L	0.067 μg/L			[93]
Heavy metal ions								
SNW1/GCE	Black Tea, Rice, Pepper, Salt	ASSWV ^i^	Pb^2+^	0.01–0.3	0.00072			[94]
			Hg^2+^	0.05–0.3	0.01211	Superior wide linear responses, low LOD; some working electrodes can enable simultaneous analysis of multiple metal ions	There are few COF-based electrode materials for heavy metal ions detection	
Fe_3_O_4_@SNW1/GCE	Red pepper powder; black tea, rice	SWASV ^j^	Pb^2+^	0.003–0.3	0.95 × 10^−3^			[95]
COF/MWCNTs/CLS/Nafion/GCE	Mushroom	SWASV	Cu^2+^	0.6–63.5 μg/L	0.2 μg/L			[96]
			Pb^2+^	2.1–207.2 μg/L	0.7 μg/L			
			Cd^2+^	1.1–112.4 μg/L	0.4 μg/L			
Fungal toxin, bacterium								
TpBD-GCE	Milk samples	DPV	Aflatoxin M1	0.5–80 μg/L	0.15 μg/L	High selectivity and sensitivity; good accuracy and speed	Limited useful electrode materials	[97]
m-COF@IgY/SPE	Milk, beef, shrimp	SWV ^k^	*E. coli*	10–10^8^ CFU/mL	3 CFU/mL			[98]

^a^: carbon cloth; ^b^: pencil graphite electrode; ^c^: screen-printed electrode; ^d^: Au electrodes; ^e^: differential pulse voltammetry; ^f^: electrochemiluminescence; ^g^: Electrochemical impedance spectroscopy; ^h^: Cyclic voltammetry; ^i^: anodic stripping square wave voltammetry; ^j^: Square wave anodic stripping voltammetry; ^k^: Square wave voltammetry; ^l^: O,O-dime-thyl-O-2,2-dichlorovinylphosphate; ^m^: limit of detection.

## Data Availability

Not applicable.
